# Supplementary material for: A junctional PACSIN2/EHD4/MICAL-L1 complex coordinates VE-cadherin trafficking for endothelial migration and angiogenesis
Source: Nat Commun. 2021 May 10;12:2610. doi: 10.1038/s41467-021-22873-y (PMC8110786; doi:10.1038/s41467-021-22873-y)
Supplement: Supplementary file 1 — Supplementary Information [file 41467_2021_22873_MOESM1_ESM.pdf]

Supplementary Figure 1

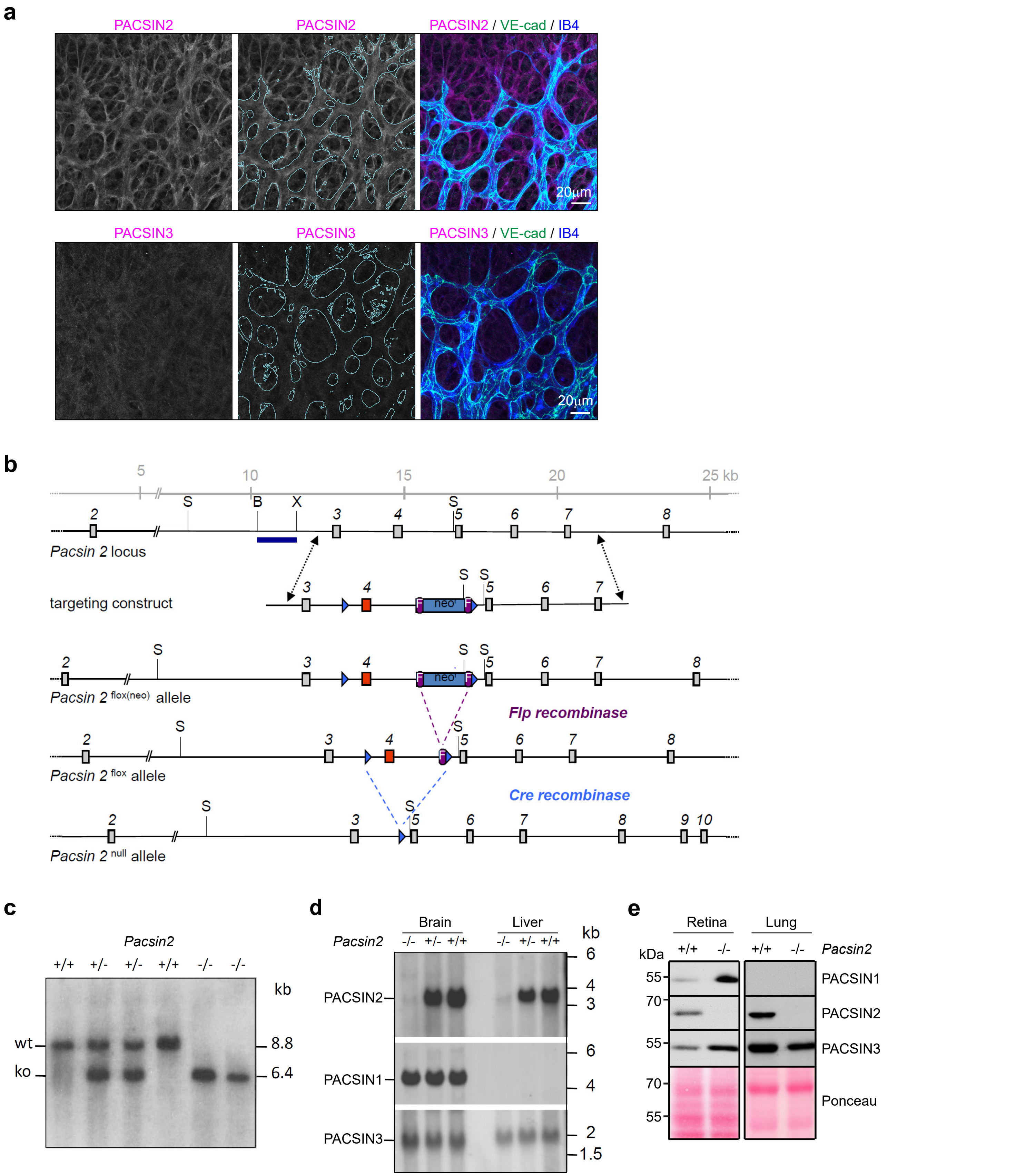

Supplementary Figure 1 - Targeting strategy and validation of conditional *Pacsin2*$^{-/-}$ mice.
(a) Representative images of wild-type P6 retinas stained for VE-cadherin (green), IB4 (blue) and PACSIN2 or PACSIN3 (magenta). Observed in at least 4 wild-type retinas from 2 independent littermates. Mask outline in blue in the middle panel corresponds to the IB4-positive vascular tissue. Scale bar – 20 µm. **(b)** Genomic map of the central part of the endogenous *Pacsin2* locus, the targeting construct, the targeted floxed allele (*Pacsin2* $^{flox(neo)}$) resulting from homologous recombination, the targeted floxed allele after removal of the neo-cassette (*Pacsin2* ^{flox}) resulting from Flp-mediated recombination, and the null allele (*Pacsin2* ^{null}) resulting from Cre-mediated recombination are depicted. Exons are indicated as numbered boxes, the floxed exon 4 corresponding to a major region of the F-BAR domain is shown in red. Triangles indicate *loxP* insertion sites. Boxes marked with an F indicate FRT insertion sites. The blue bar indicates the probe used for Southern hybridization. Restriction sites are: B = BamHI; S = ScaI; X = XhoI. **(c)** Southern blot analysis. *Pacsin2*$^{+/+}$, $^{+/-}$ and $^{-/-}$ mice were identified by digestion of genomic DNA with ScaI. Fragments were separated according to size and hybridized with a BamHI/XhoI DNA fragment indicated in (b) serving as 5'-external probe. **(d)** Northern blot analysis. Poly (A)+ RNA was isolated from brain (left panel) and liver (right panel) from mice of the indicated genotypes. The transcripts were separated according to size and hybridized to the indicated *Pacsin* isoform specific probes. **(e)** Expression of PACSIN proteins in retina and lung tissue. Western blot analysis of PACSIN1, PACSIN2 and PACSIN3 in lysates from retina and lung tissue derived from *Pacsin2*$^{+/+}$ or *Pacsin2*$^{-/-}$ mice. Southern, Northern and Western blots were repeated 3 times with similar results. WT – wild type, KO – knock out. Source data are provided as a Source Data file.

Supplementary Figure 2

a

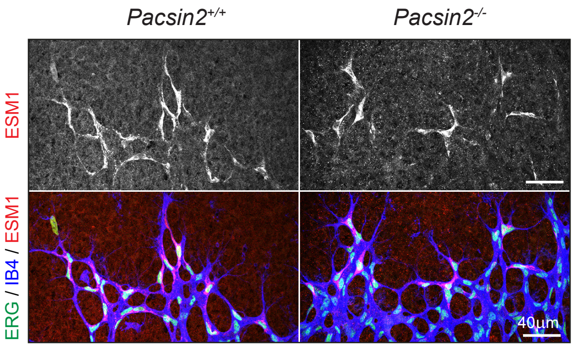

b

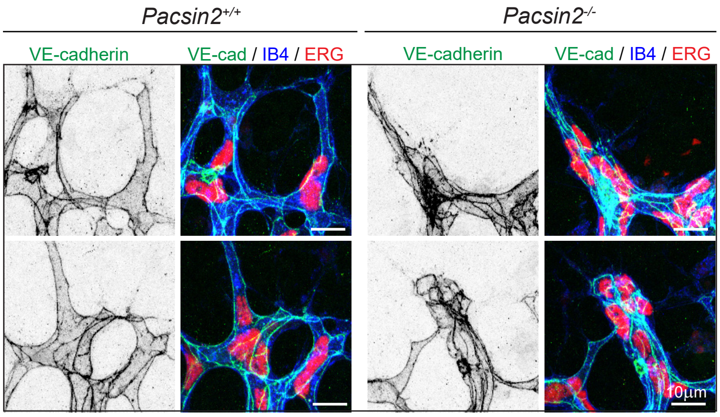

Supplementary Figure 2 - Characterization of angiogenic sprouts in *Pacsin2*$^{-/-}$ retinal vasculature.
(a) Representative images of the vascular sprouting front of P6 control and *Pacsin2*$^{-/-}$ retinas stained for the endothelial tip cell marker ESM1 (red), ERG (green) and IB4 (blue). Observed in at least 4 retinas per genotype from 2 independent littermates. **(b)** High magnification images of representative sprouts from control and *Pacsin2*$^{-/-}$ P6 retinas stained for VE-cadherin (green), IB4 (blue) and ERG (red). Observed in at least 7 retinas per genotype from 2 independent littermates. Scale bar – 40 µm and 10 µm.

Supplementary Figure 3

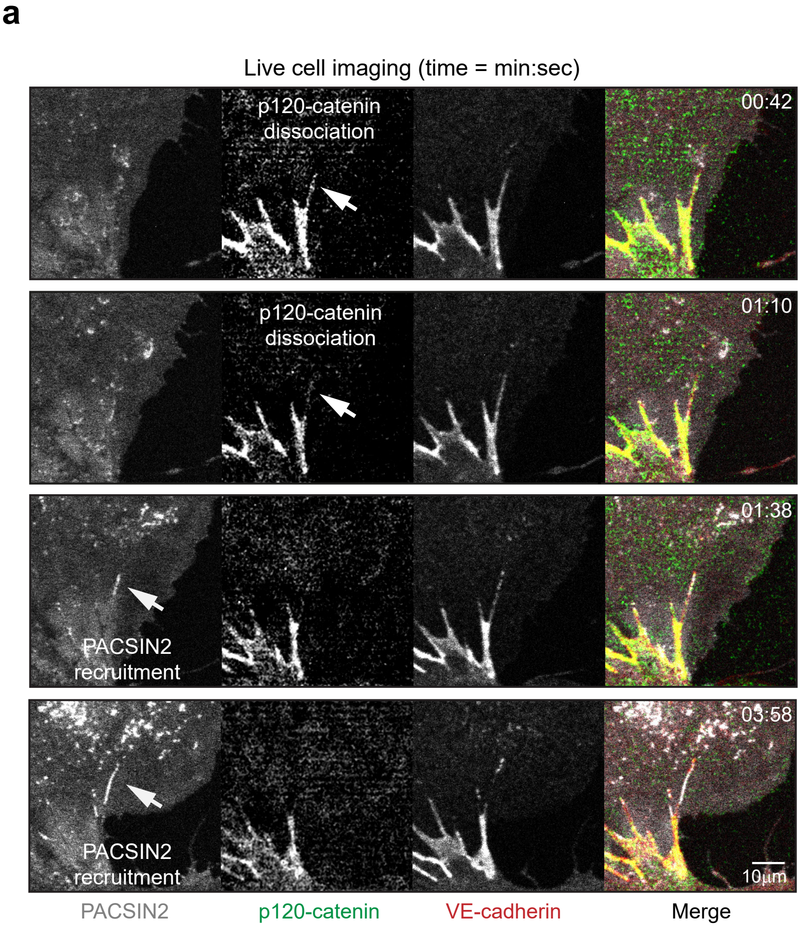

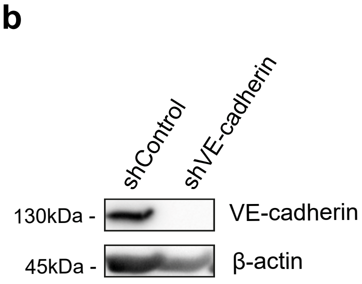

Supplementary Figure 3 - P120-catenin dissociation from asymmetric AJs precedes PACSIN2 recruitment.
(a) Stills from a 4 min time-lapse confocal recordings of asymmetric AJs formed by HUVECs expressing PACSIN2-GFP (greyscale) and p120-catenin-mCherry (green) that were live-labelled with anti-VE-cadherin antibody (red). See Supplementary Movie 2 for the corresponding time-lapse movie. Scale bar - 10 µm. **(b)** Representative Western blot analysis of VE-cadherin and β-actin (loading control) protein levels in whole-cell lysates from HUVECs transduced with shControl and shVE-cadherin-3'UTR. Western blots were repeated 3 times with similar results. Source data are provided as a Source Data file.

Supplementary Figure 4

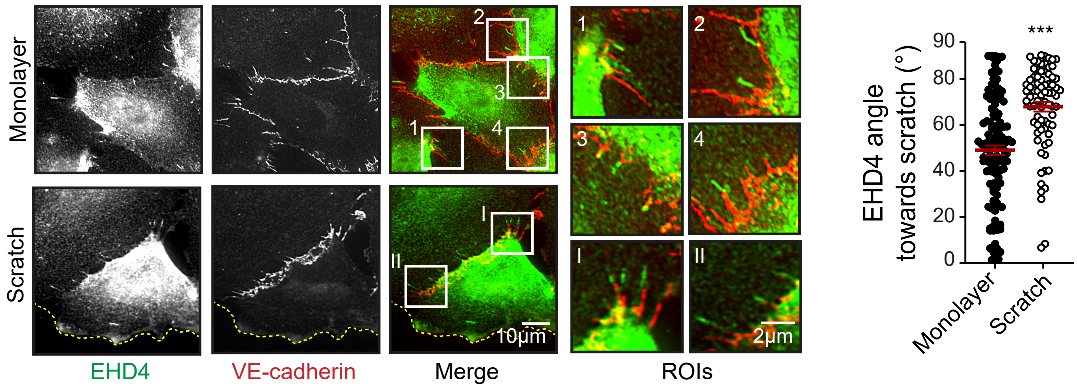

Supplementary Figure 4 - EHD4 is recruited to the trailing ends of asymmetric AJs in directionally migrating HUVECs.
Widefield IF images of HUVEC monolayer (upper panel) or HUVEC migrating towards a scratch wound and stained for EHD4 (green) and VE-cadherin (red). The yellow punctuated line indicates the boundaries of the scratch wound induced 5 h prior to fixation. The white squares indicate ROIs that are magnified in the right panels. The graph shows the quantified orientation of EHD4-positive asymmetric AJs in relation to the scratch or the bottom edge of the image (for the normal monolayer condition). The graph represents mean ± SEM (error bars). Statistical analysis was performed by an unpaired two-tailed t-test. $P<0.0001$ when comparing monolayer to scratch wound conditions. Data from 3 independent experiments; monolayer (n=125 EHD4-positive asymmetric AJs) and scratch (n=76 EHD4-positive asymmetric AJs). ***$P < 0.001$. Scale bar – 10 and 2 μm. ROIs – regions of interest, AJ - adherens junction. Source data are provided as a Source Data file.

Supplementary Figure 5

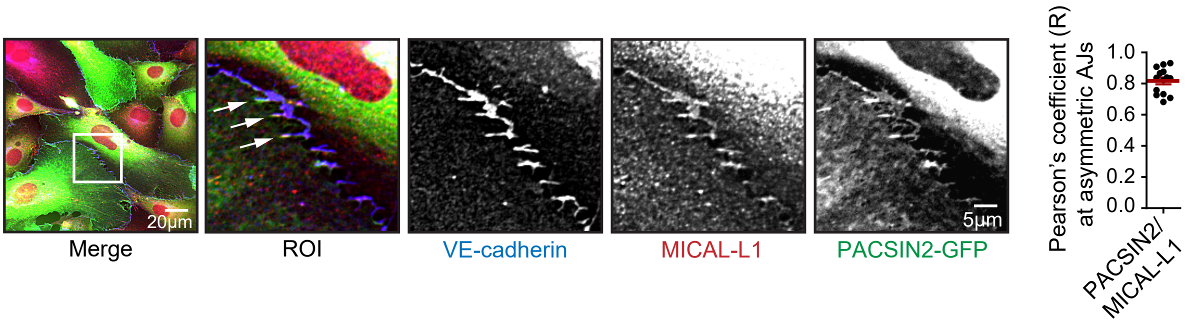

Supplementary Figure 5 - Colocalization between PACSIN2 and MICAL-L1 at asymmetric AJs.
Widefield IF images of HUVECs expressing PACSIN2-GFP (green) and stained for MICAL-L1 (red) and VE-cadherin (blue). The white arrows indicate colocalization between MICAL-L1 and PACSIN2. The graph shows Pearson's correlation analysis of fluorescent signals of PACSIN2 and MICAL-L1 at the asymmetric AJs. Data is from 3 independent experiments and n=14 MICAL-L1-positive asymmetric AJs. The graph represents mean ± SEM (error bars). Scale bars - 20 and 5 µm. ROI – region of interest, AJ - adherens junction. Source data are provided as a Source Data file.

Supplementary Figure 6

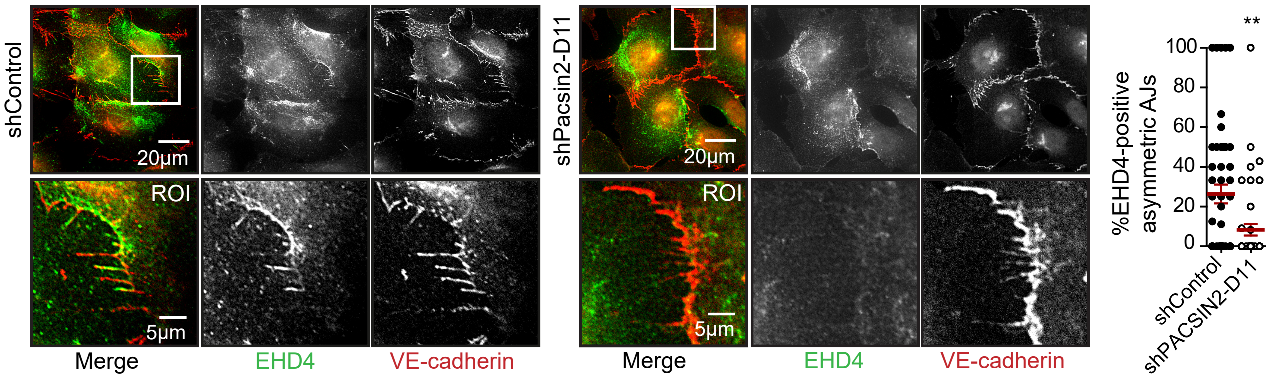

Supplementary Figure 6 - Depletion of PACSIN2 from HUVECs perturbs recruitment of EHD4 to asymmetric AJs.
Widefield IF images of HUVECs transduced with shControl or shPACSIN2-D11 and stained for EHD4 (green) and VE-cadherin (red). The graph represents the percentage of EHD4-positive asymmetric AJs in HUVECs transduced with shControl (n=45 endothelial cells) or shPACSIN2-D11 (n=42 endothelial cells) from 3 independent experiments. P=0.0016 when comparing shControl to shPACSIN2-D11. The graph represents mean ± SEM (error bars). The statistical analysis was performed on by an unpaired two-tailed t-test. **P < 0.01. Scale bar – 20 and 5 μm. ROI – region of interest, AJ - adherens junction. Source data are provided as a Source Data file.

Supplementary Figure 7

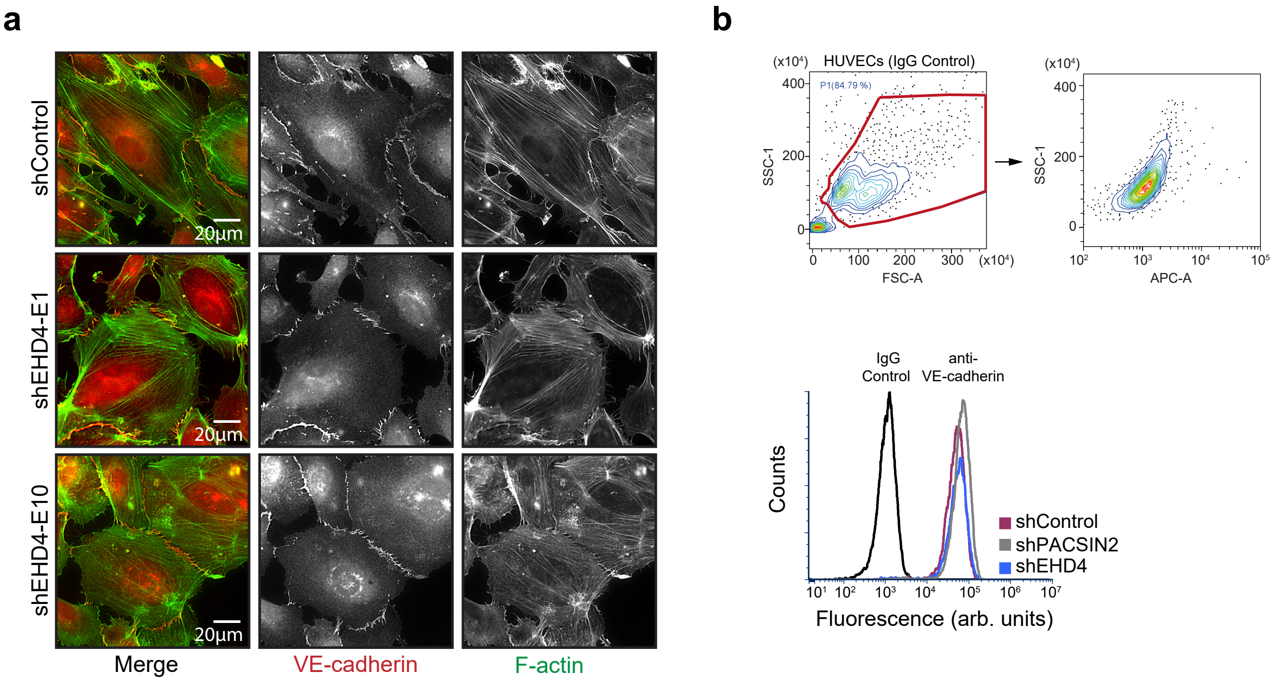

Supplementary Figure 7 - Depletion of EHD4 from HUVECs does not cause major phenotypic changes or affect VE-cadherin surface levels.
(a) Widefield IF images of HUVECs transduced with shControl, shEHD4-E1 or shEHD4-E10 and stained for VE-cadherin (red) and F-actin (green). Imaging experiments were repeated 3 times with similar results. Scale bar – 20 µm. **(b)** Upper panel: gating selection to determine the level of VE-cadherin expression on HUVEC cells (IgG control) by flow cytometry. Lower panel: Representative flow cytometry analysis of VE-cadherin surface expression on HUVECs transduced with shControl, shPACSIN2 or shEHD4 (10.000 counted cells per condition). Plots are representatives from 2 independent experiments. Arb. units – arbitrary units.

Supplementary Figure 8

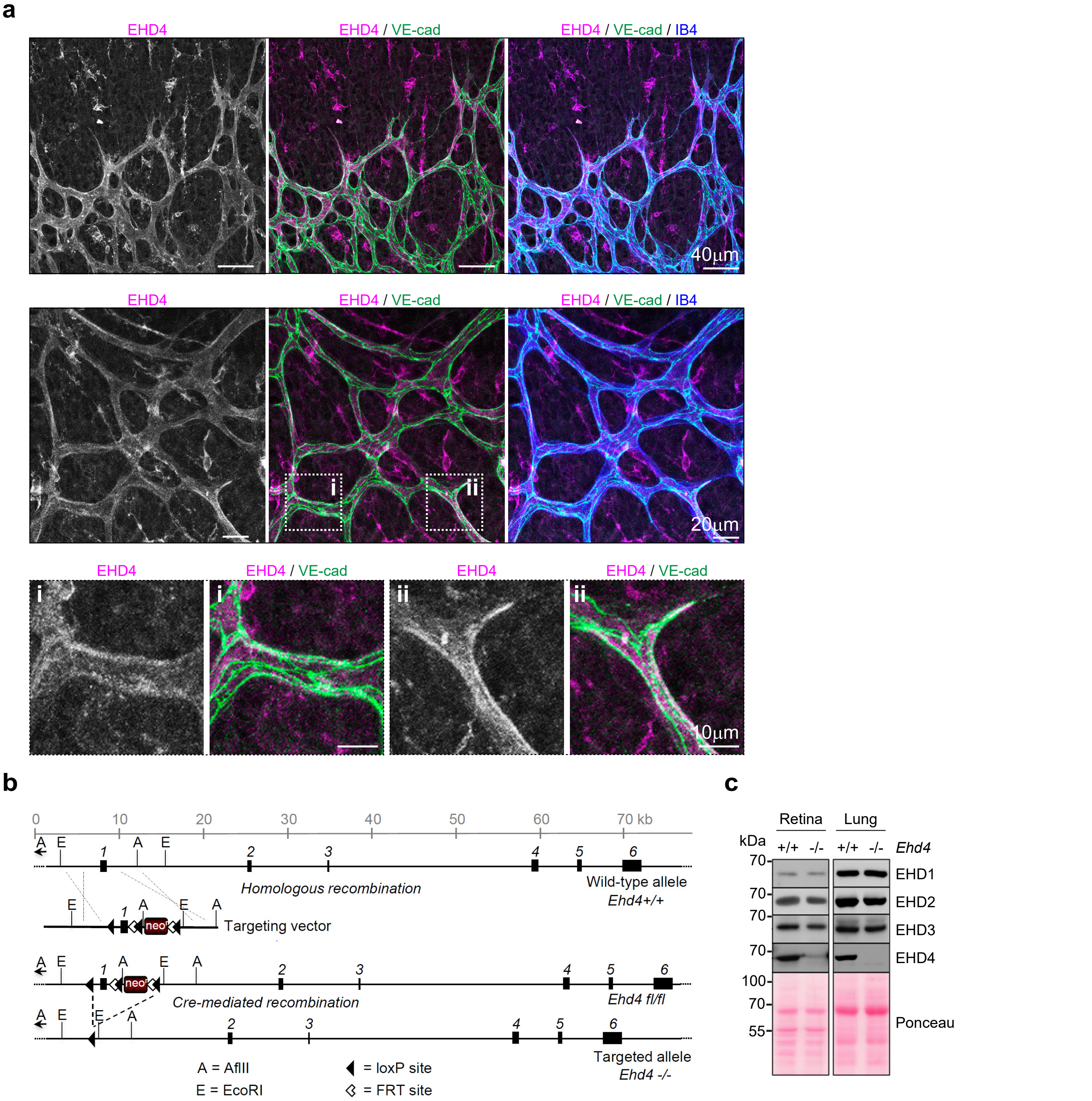

Supplementary Figure 8 - Targeting strategy and validation of conditional *Ehd4*$^{-/-}$ mice.
(a) Representative images of wild-type P6 retinas stained for EHD4 (magenta), VE-cadherin (green), IB4 (blue) at different magnifications. Observed in at least 4 wild-type retinas from 2 independent littermates. Mask outline in blue in the middle panel corresponds to the IB4-positive vascular tissue. Scale bar – 40, 20 or 10 µm. **(b)** Schematic depiction of the *Ehd4* targeting strategy. The wild-type *Ehd4* allele is shown with restriction enzyme sites (A, AflII; E, EcoRI) and the location of exons 1–6 (black boxes). The restriction sites were used to analyze the recombinants. The targeting vector was generated by introducing a single *loxP* site 5' and a *neo* gene cassette flanked by FRT and *loxP* sites 3' of exon 1. The location of the *loxP* and FRT sites are indicated. Homologous recombination led to the floxed *Ehd4* allele (*Ehd4* $^{fl/fl}$) and Cre-mediated recombination to the targeted *Ehd4* allele *Ehd4*$^{-/-}$. **(c)** Western blot analysis of EHD1, EHD2, EHD3 and EHD4 in lysates from retina and lung tissue derived from *Ehd4*$^{-/-}$ or wild-type control mice. Western blots were repeated 3 times with similar results. Source data are provided as a Source Data file.

Supplementary Table 1: Primer sequences

Name	Sequence (5'-3')
shVE-cadherin-3’UTR	ccggtGGATAGCAAACTCCAGGTTCCctcgagGGAACCTGGAGTTTGCTATCCtttttg
eGFP 5'end	gagatctagaATGGTGAGCAAGGGCGA
EHD4 3'end	gagagctagcTCAGTCGGCCTTGGGCA
VEC DEE 646-648 AAA fwd	CTGGTCACCTACGCAGCAGCAGGCGGCGGCGAGATG
VEC DEE 646-648 AAA rev	CATCTCGCCGCCGCCTGCTGCTGCGTAGGTGACCAG
VEC GGG 649-651 AAA fwd	TACGACGAGGAGGCAGCAGCAGAGATGGACACC
VEC GGG 649-651 AAA rev	GGTGTCCATCTCTGCTGCTGCCTCCTCGTCGTA
