# Supplementary material for: A junctional PACSIN2/EHD4/MICAL-L1 complex coordinates VE-cadherin trafficking for endothelial migration and angiogenesis
Source: Nat Commun. 2021 May 10;12:2610. doi: 10.1038/s41467-021-22873-y (PMC8110786; doi:10.1038/s41467-021-22873-y)
Supplement: Supplementary file 3 — Description of Additional Supplementary Files [file 41467_2021_22873_MOESM3_ESM.pdf]

Description of Additional Supplementary Files

File Name: Supplementary Movie 1
Description: **PACSIN2 is needed for endothelial directional migration.** Time lapse recording of HUVECs transduced with shControl, shPACSIN2-D11 or shPACSIN2-E1 during scratch wound migration. Images were acquired by time-lapse phase-contrast microscopy (NIKON Eclipse Ti) using a 10x dry objective. Frames were taken every 10 min for ~ 9 h.

File Name: Supplementary Movie 2
Description: **p120-catenin dissociation from asymmetric AJs precedes PACSIN2 recruitment.** Time lapse recording of HUVECs expressing PACSIN2-GFP (greyscale), p120-catenin-mCherry (green) and live-labelled with anti-VE-cadherin antibody (red). Images were acquired by confocal microscopy (Leica TCS SP8 SMD) using a 63x/1.4 NA oil objective. Frames were taken every 14 sec for 9 min.

File Name: Supplementary Movie 3
Description: **EHD4 is recruited to the trailing end of asymmetric AJs.** Time lapse recording of HUVECs transduced with EHD4-GFP (green) and VE-cadherin-mCherry (red). Images were acquired by time-lapse widefield microscopy (NIKON Eclipse Ti) using a 60x 1.49 NA oil objective. Frames were taken every 15 sec for 10 min.

File Name: Supplementary Movie 4
Description: **EHD4 depletion perturbs the turnover of asymmetric AJs.** Time lapse recording of shControl and shEHD4 HUVECs transduced with VE-cadherin-GFP. Movie highlights the failure of EHD4-depleted cells to gradually turnover the asymmetric AJs. Images were acquired by time-lapse widefield microscopy (NIKON Eclipse Ti) using a 60x 1.49 NA oil objective. Frames were taken every 45 sec for 64 min.

File Name: Supplementary Movie 5
Description: **EHD4 depletion perturbs asymmetric AJ remodeling during collective migration.** Time lapse recording of HUVECs transduced with shControl or shEHD4 and live-labelled with anti-VE-cadherin antibody during scratch wound migration. Movie shows the formation of longer asymmetric AJs that have longer lifetime in EHD4-depleted HUVECs. Images were acquired by time-lapse widefield microscopy (NIKON Eclipse Ti) using a 60x 1.49 NA oil objective. Frames were taken every 30 sec for 55 min.

File Name: Supplementary Movie 6
Description: **EHD4 is needed for endothelial directional migration.** Time lapse recording of HUVECs transduced with shControl, shEHD4-E1 or shEHD4-E10 during scratch wound migration. Images were acquired by time-lapse phase-contrast microscopy (NIKON Eclipse Ti) using a 10x dry objective. Frames were taken every 10 min for ~ 8 h.
