# Supplementary material for: A junctional PACSIN2/EHD4/MICAL-L1 complex coordinates VE-cadherin trafficking for endothelial migration and angiogenesis
Source: Nat Commun. 2021 May 10;12:2610. doi: 10.1038/s41467-021-22873-y (PMC8110786; doi:10.1038/s41467-021-22873-y)
Supplement: Supplementary file 10 — Reporting Summary [file 41467_2021_22873_MOESM10_ESM.pdf]

nature research

Corresponding author(s): Stephan Huveneers

Last updated by author(s): Mar 12, 2021

Reporting Summary

Nature Research wishes to improve the reproducibility of the work that we publish. This form provides structure for consistency and transparency in reporting. For further information on Nature Research policies, see Authors & Referees and the Editorial Policy Checklist .

Statistics

For all statistical analyses, confirm that the following items are present in the figure legend, table legend, main text, or Methods section.

n/a	Confirmed	
☐	☒	The exact sample size (*n*) for each experimental group/condition, given as a discrete number and unit of measurement
☐	☒	A statement on whether measurements were taken from distinct samples or whether the same sample was measured repeatedly
☐	☒	The statistical test(s) used AND whether they are one- or two-sided<br>*Only common tests should be described solely by name; describe more complex techniques in the Methods section.*
☒	☐	A description of all covariates tested
☐	☒	A description of any assumptions or corrections, such as tests of normality and adjustment for multiple comparisons
☐	☒	A full description of the statistical parameters including central tendency (e.g. means) or other basic estimates (e.g. regression coefficient) AND variation (e.g. standard deviation) or associated estimates of uncertainty (e.g. confidence intervals)
☐	☒	For null hypothesis testing, the test statistic (e.g. *F*, *t*, *r*) with confidence intervals, effect sizes, degrees of freedom and *P* value noted<br>*Give P values as exact values whenever suitable.*
☒	☐	For Bayesian analysis, information on the choice of priors and Markov chain Monte Carlo settings
☒	☐	For hierarchical and complex designs, identification of the appropriate level for tests and full reporting of outcomes
☐	☒	Estimates of effect sizes (e.g. Cohen's *d*, Pearson's *r*), indicating how they were calculated

Our web collection on statistics for biologists contains articles on many of the points above.

Software and code

Policy information about availability of computer code

Data collection	Nikon Imaging Software Elements 5.02.03 (Nikon), Leica Application Suite X 3.7.1.21655 (Leica), ImageQuant LAS4000 mini V1.3 (Fuji), EVOS M7000 (Thermofisher)
Data analysis	Prism Graphpad V8.03, Image J (FIJI open source version 1.53c), Chemotaxis tool 2.0 Image J plugin, Adobe Photoshop CS6, MATLAB (V9.6.0), PIVlab software version 2.40 using MatPIV (https://www.mn.uio.no/math/english/people/aca/jks/matpiv/).

For manuscripts utilizing custom algorithms or software that are central to the research but not yet described in published literature, software must be made available to editors/reviewers. We strongly encourage code deposition in a community repository (e.g. GitHub). See the Nature Research guidelines for submitting code & software for further information.

Data

Policy information about availability of data

All manuscripts must include a data availability statement. This statement should provide the following information, where applicable:

- Accession codes, unique identifiers, or web links for publicly available datasets
- A list of figures that have associated raw data
- A description of any restrictions on data availability

Source image data underlying Figs. 2a, 4d, 4f and 5b, and Supplementary Figs. 1c, 1d, 1e, 3b and 8c are provided as a Source Data File. The numerical data underlying Figs. 1c, 1d, 1f, 2c, 2e, 2f, 2h, 2i, 2l, 2n, 2o, 3c, 3d, 3g, 4c, 4e, 4g, 4j, 4k, 4l, 5b, 5d, 5f, 6b, 6f, 6g, 7b, 7c, 7e, 7g, 7i and 7k, and Supplementary Figs. 4, 5, and 6 are provided as a Source Data File. Additional datasets generated in the current study are available from the corresponding author on request.

Field-specific reporting

Please select the one below that is the best fit for your research. If you are not sure, read the appropriate sections before making your selection.

- [x] Life sciences
- [ ] Behavioural & social sciences
- [ ] Ecological, evolutionary & environmental sciences

For a reference copy of the document with all sections, see nature.com/documents/nr-reporting-summary-flat.pdf

Life sciences study design

All studies must disclose on these points even when the disclosure is negative.

Sample size	Sample size and experimental replicates are indicated in the figure legends. For the animal experiments, we based the group sample size on power calculations using parameters established in previous studies (PMID: 30446640, 29449337). This is in line with the typical group size (5-8 mice) that is used by others in the field and is appropriate for the experimental setup. For other experiments Sample sizes were not statistically predetermined but were chosen based on previous experience, standards in the fields and previously published literature.
Data exclusions	No data was excluded from the analysis.
Replication	All findings in the study have been successfully reproduced by independent experiments. See experimental details in figure legends.
Randomization	No randomization was used. Experimental groups were allocated based on genotype and gene silencing.
Blinding	Part of the image acquisition and analysis of retina's from mice were performed in a blinded manner. The rest of the study is based on multiple types of experiments, performed separately by independent researchers, quantified over independent experiments and hence were not performed in a blinded manner.

Reporting for specific materials, systems and methods

We require information from authors about some types of materials, experimental systems and methods used in many studies. Here, indicate whether each material, system or method listed is relevant to your study. If you are not sure if a list item applies to your research, read the appropriate section before selecting a response.

Materials & experimental systems

n/a	Involved in the study	
[ ]	[x]	Antibodies
[ ]	[x]	Eukaryotic cell lines
[x]	[ ]	Palaeontology
[ ]	[x]	Animals and other organisms
[x]	[ ]	Human research participants
[x]	[ ]	Clinical data

Methods

n/a	Involved in the study	
[x]	[ ]	ChIP-seq
[x]	[ ]	Flow cytometry
[x]	[ ]	MRI-based neuroimaging

Antibodies

Antibodies used

Rabbit polyclonal anti-human PACSIN2 (Cat # AP8088b, Abgent)
Mouse anti-human p120-catenin (Clone 98/pp120 (RUO), Cat # 610134, BD Biosciences)
Goat anti-human VE-cadherin (Clone C-19, Cat # SC-6458, Santa Cruz)
Rabbit polyclonal anti-human VE-cadherin (Cat # 160840, Cayman Chemical)
Mouse anti-cadherin-5 (Clone 75, Cat # 610252, BD Biosciences)
Rat anti-mouse VE-cadherin (# 555289, BD Bioscience)
Mouse anti-human CD144 Alexa Fluor 647 (Clone 55-7H1; Cat # 561567; BD Biosciences)
Rabbit monoclonal anti-ERG (Clone EPR3864, # AB92513, Abcam)
Rabbit anti-human MICAL-L1 (Cat # NBP2-55389, Novus Biologicals)
Goat polyclonal anti-endocan/ESM1 (Cat # AF1999, from R&D Systems)
Mouse anti-GM130 (clone 35, Cat # 610823, BD Biosciences)
Rabbit polyclonal anti-GOLPH4/GPP130 (Cat # ab28049, Abcam)
Rat anti-ICAM2 (Clone 3C4(mIC2/4), Cat # 553326, BD Pharmingen)
Mouse monoclonal anti-human vinculin (Clone hVIN-1, Cat # V9131-100UL, Sigma Aldrich)
Mouse anti-GFP (Clone B-2, Cat # sc-9996, Santa Cruz)
Rabbit anti EHD1, EHD2, EHD3, EHD4, PACSIN1, PACSIN2 and PACSIN3 were a gift from the laboratory of Dr Markus Plomann.
Isolectin GS-IB4 Alexa Fluor 488 (Invitrogen, I21411)
Isolectin GS-IB4 Alexa Fluor 568 (Invitrogen, I21412)

Isolectin GS-IB4 Alexa Fluor 647 (Invitrogen, I32450)
Chicken anti mouse Alexa Fluor 488 (Invitrogen, A21200)
Chicken anti-rabbit Alexa Fluor 488 (Invitrogen, A21441)
Chicken anti-mouse Alexa Fluor 594 (Invitrogen, A21201)
Chicken anti-rabbit Alexa Fluor 594 (Invitrogen, A21442)
Chicken anti-mouse Alexa Fluor 647 (Invitrogen, A21463)
Chicken anti-rabbit Alexa Fluor 647 (Invitrogen, A21443)
Chicken anti-goat Alexa Fluor 647 (Invitrogen, A21469)
Goat anti-rabbit Alexa Fluor 488 (Invitrogen, A11008)
Goat anti-rabbit Alexa Fluor 568 (Invitrogen, A11011)
Goat anti-rat Alexa Fluor 488 (Invitrogen, A11006)
Goat anti-rat Alexa Fluor 633 (Invitrogen, A21094)
Donkey anti-rabbit IgG (Jackson ImmunoResearch, 711-006-152)
Goat Anti-mouse-HRP (Invitrogen, A28177)
Goat Anti-rabbit-HRP (Invitrogen, A27036)

Validation

The antibodies were validated by us in the current manuscript, by the commercial providers and additionally in our previous studies (PMID: 30446640, 27417273, 22391038, 32755570, 31964713, 31268670). Specifically, in Figure 2a of this manuscript we have validated the specificity of the rabbit anti-human PACSIN2 antibody (Abgent, AP8088b) for Western blotting of human PACSIN2 in lysates of shControl and shPACSIN2 HUVECs, and the antibody is validated by manufacturer Abgent for Western blot analysis and immunohistochemistry. In Figure 5b of this manuscript we have validated the specificity of the rabbit anti-EHD4 antibody (gift Markus Plomann) for Western blotting of human EHD4 in lysates of shControl and shEHD4 HUVECs. In Supplementary Figure 1e of this manuscript we have validated the specificity of the rabbit anti-PACSIN2 antibody (gift Markus Plomann) for Western blotting of mouse PACSIN2 in lysates of tissues from control and PACSIN2-/- knock out mice. In Supplementary Figure 8c of this manuscript we have validated the specificity of the rabbit anti-EHD4 antibody (gift Markus Plomann) for Western blotting of mouse EHD4 in lysates of tissues from control and EHD4-/- knock out mice. Mouse anti-human p120-catenin antibody (Clone 98/pp120, BD Biosciences, 610134) was validated by the manufacturer BD Biosciences for Western blot, immunohistochemistry, immunoprecipitation, and immunofluorescence in human cells and has been used previously for endothelial cells by Lampugnani et al. (PMID: 11950930). Goat anti-human VE-cadherin (Clone C-19, Santa Cruz SC-6458) was validated by the manufacturer Santa Cruz for Western blot analysis and has been used previously for immunofluorescence and Western blot analysis in endothelial cells by Dorland et al. (PMID: 27417273). Rabbit anti-human VE-cadherin antibody (Cayman Chemical, 160840) was validated by the manufacturer Cayman Chemical for Western blot analysis, immunoprecipitation and Immunocytochemistry, and has been used previously for endothelial cells by Breviario et al. (PMID: 7627717) and Dorland et al. (PMID: 27417273). Mouse anti-cadherin-5 antibody (Clone 75, BD Biosciences, 610252) was validated by the manufacturer BD Biosciences for human VE-cadherin in Western blot analysis, immunofluorescence and immunohistochemistry, and has been used previously for endothelial cells by Huveneers et al. (PMID: 22391038) and Dorland et al. (PMID: 27417273). Rat anti-mouse VE-cadherin antibody (BD Bioscience, 555289) was validated by the manufacturer BD Biosciences for flow cytometry, immunohistochemistry and immunoprecipitation, and has been used previously for endothelial cells by Angulo-Urarte et al. (PMID: 30446640). Mouse anti-human CD144 Alexa Fluor-647 antibody (Clone 55-7H1; BD Biosciences, 561567) has been validated by manufacturer BD Biosciences for use in flow cytometry and has been used for endothelial cells by Dorland et al. (PMID: 27417273) and Kroon et al. (PMID: 25146919). Rabbit anti-ERG antibody (Clone EPR3864, Abcam, AB92513) has been validated by manufacturer Abcam for mouse, rat and human ERG in Western blot analysis, immunohistochemistry, immunofluorescence and flow cytometry, and has been used for endothelial cells by Angulo-Urarte et al. (PMID: 30446640) and Carvalho et al. (PMID: 31246175). Rabbit anti-human MICAL-L1 (Novus Biologicals, NBP2-55389) has been validated by manufacturer Novus Biologicals for immunofluorescence and has been used by Giridhadan et al. (PMID: 23596323). Goat anti-endocan/ESM1 antibody (R&D Systems, AF1999) has been validated by manufacturer R&D systems for mouse ESM1 in ELISAs and Western blot analysis, and has been used for endothelial cells by Rocha et al. (PMID: 25057127). Mouse anti-GM130 antibody (clone 35, BD Biosciences, 610823) has been validated by manufacturer BD Biosciences for rat, human and mouse in Western blot analysis, immunofluorescence and immunoprecipitation, and has been used for endothelial cells by Franco et al. (PMID: 26845523). Rabbit anti-GOLPH4/GPP130 antibody (Abcam, ab28049) has been validated for immunohistochemistry in mouse, rat, human by manufacturer Abcam, and has been used for endothelial cells by Franco et al. (PMID: 25884288). Rat anti-ICAM2 antibody (Clone 3C4(mIC2/4); BD Pharmingen, 553326) has been validated by manufacturer BD Pharmingen for mouse in flow cytometry, immunohistochemistry and immunoprecipitation, and has been used for endothelial cells by Franco et al. (PMID: 25884288). Mouse anti-human vinculin antibody (Clone hVIN-1; Sigma Aldrich, V9131-100UL) has been validated by the manufacturer Sigma Aldrich for mouse, rat and human in immunohistochemistry, immunofluorescence and Western blot analysis, and has been used for endothelial cells by Huveneers et al. (PMID: 22391038). Mouse anti-GFP antibody (Clone B-2, Santa Cruz, sc-9996) has been validated by the manufacturer Santa Cruz for Western blot anlaysis, immunoprecipitation, immunofluorescence, flow cytometry and ELISA, and has been used for endothelial cells by van der Stoel et al. (PMID: 31964713). Rabbit anti-EHD1, EHD2, EHD3, PACSIN1 and PACSIN3 antibodies were produced by the lab of Markus Plomann and validated using recombinant proteins and knockout models, and has been used for Western blot analysis and immunofluorescence by Modregger et al. (PMID: 11082044) and Blume et al. (PMID: 17097635).

Eukaryotic cell lines

Policy information about cell lines

Cell line source(s)

Primary human umbilical vein endothelial cells (HUVEC) pooled from different donors were obtained from Lonza. HEK293T cells were obtained from ATCC.

Authentication	The endothelial nature of HUVECs were confirmed by VE-cadherin stainings, and cultured up to passage 5 to prevent differentiation. HEK293T cells were not authenticated.
Mycoplasma contamination	Cell lines tested negative for mycoplasma contamination.
Commonly misidentified lines (See ICLAC register)	No misidentified lines were used in the study.

Animals and other organisms

Policy information about studies involving animals; ARRIVE guidelines recommended for reporting animal research

Laboratory animals	In the study newborn Pacsin2-/- and Ehd4-/- C57BL/6 male and female mice were analyzed at postnatal day 6. Animals were housed in the Center for Molecular Medicine animal care facility of the University of Cologne under standard pathogen-free, temperature- and humidity-controlled conditions with a 12-h light/dark schedule and provided with food and water ad libitum.
Wild animals	No wild animals were used in the study.
Field-collected samples	No field-collected samples were used in the study.
Ethics oversight	All mouse studies were performed after ethical approval for breeding and maintaining mice by local government authorities (Landesamt für Natur, Umwelt und Verbraucherschutz Nordrhein-Westfalen (LANUV; permission number no. 81-02.04.2019A215, Germany) in accordance with the German animal protection law and with permission to sacrifice mice for scientific purpose. Experimental procedures were performed according to all relevant ethical regulations for animal testing and research.

Note that full information on the approval of the study protocol must also be provided in the manuscript.
